# Pooled prevalence and multilevel determinants of stillbirths in sub-Saharan African countries: implications for achieving sustainable development goal

**DOI:** 10.1186/s41256-024-00395-6

**Published:** 2025-02-27

**Authors:** Bewuketu Terefe, Mahlet Moges Jembere, Nega Nigussie Abrha, Dejen Kahsay Asgedom, Solomon Keflie Assefa, Nega Tezera Assimamaw

**Affiliations:** 1 Department of Community Health Nursing, School of Nursing, College of Medicine and Health Sciences, University of Gondar, Gondar, Ethiopia; 2 Department of Emergency, and Critical Care Nursing, School of Nursing, College of Medicine and Health Sciences, University of Gondar, Gondar, Ethiopia; 3 Department of Public Health, College of Medicine and Health Sciences, Samara University, Afar, Ethiopia; 4 Pawe Health Science College, Pawe, Northwest Ethiopia Ethiopia; 5 Department of Epidemiology, and Biostatistics, Institute of Public Health, College of Medicine and Health Sciences, University of Gondar, Gondar, Ethiopia; 6 Department of Pediatrics, and Child Health Nursing, School of Nursing, College of Medicine and Health Sciences, University of Gondar, Gondar, Ethiopia

**Keywords:** Determinants, Stillbirth, Sub-Saharan Africa, Sustainable development goal, Multilevel modeling

## Abstract

**Background:**

Despite being included in the Millennium Development Goals (MDGs) and now the Sustainable Development Goals (SDGs), stillbirths remain overlooked with limited regional research, highlighting an ongoing gap in addressing this issue. However, a staggering 2 million stillbirths occur each year, equivalent to one every 16 s. Furthermore, approximately 98% of these stillbirths take place in developing countries, particularly in sub-Saharan Africa (SSA). In light of these statistics and the need to address the lack of data, methodological approaches, and population gaps, this study aims to assess the prevalence and determinants of stillbirths in SSA from 2016 to 2023, aligning with the SDGs.

**Methods:**

This study used data from the Demographic and Health Survey (DHS) conducted in SSA. The analysis included a weighted sample of 212,194 pregnancies of at least 28 weeks' gestation collected from 2016 to 2023, using R-4.4.0 software. Descriptive data, such as frequencies, were performed. Stillbirth prevalence was visualized using a forest plot. A multilevel modeling analysis was used by considering individual-level factors and community level factors. The multilevel model was employed to account for clustering within countries and allow for the examination of both fixed and random effects that influence stillbirths. For the multivariable analysis, variables with a *p* value ≤ 0.2 in the bivariate analysis were considered. The Adjusted Odds Ratio (AOR) with a 95% Confidence Interval (CI) and a *p* value < 0.05 were reported to indicate the statistical significance and the degree of association in the final model.

**Results:**

The pooled prevalence of stillbirths was found to be 1.54% per 100 [95% CI 1.19-2.01]. Factors positively associated with stillbirths in SSA included maternal age (25–34 years, 35–49 years), marital status (married, divorced or widowed), antenatal care visits, age at first birth (before age 20), short birth intervals, long birth intervals, birth order (second or third), residence in rural areas, country income level (lower middle income), and low literacy rate. Factors negatively associated with stillbirth mortality included maternal education (primary education, secondary or higher education), wealth index (higher economic status), access to mass media, access to improved drinking water, distance to health facilities, and country income level (upper middle income).

**Conclusions:**

Stillbirth rates fall significantly short of achieving Every Newborn Action Plan target by 2030 in SSA. The analysis of factors that affect stillbirth mortality reveals important connections. It is essential to improve maternal education, economic status, and healthcare infrastructure to decrease stillbirth rates and enhance the health outcomes of mothers and children in the region. To effectively address these risks, efforts should concentrate on increasing access to antenatal care, raising awareness, and improving socio-economic conditions. By improving access to healthcare and education, these disparities could potentially lead to a decrease in stillbirth rates in the region.

## Introduction

A stillbirth is defined as the death of a baby occurring after 28 weeks of pregnancy, either before or during birth. Shockingly, nearly 2 million stillbirths occur each year, which equates to approximately one stillbirth every 16 s [1]. Every Newborn Action Plan (ENAP) has established a worldwide goal of reducing late stillbirths to ≤ 12 per 1,000 births by 2030. As of 2021, 139 high-income countries have achieved this target. However, 56 countries are not projected to reach it without additional intervention. If present patterns persist, it is estimated that there will be 15.9 million stillbirths worldwide by 2030, with almost half of them (7.7 million, or 48%) occurring in sub-Saharan Africa (SSA) [2]. Furthermore, since stillbirths are the hidden global mortality, the United Nations International Children's Emergency Fund (UNICEF) emphasizes the urgency of addressing stillbirths, especially in low- and middle-income countries where the majority of these cases occur [3].

In SSA, the burden of stillbirths is alarmingly high. According to the Lancet Stillbirth Series (2016), more than 2 million stillbirths occur globally each year, with more than half occurring in SSA [4]. This region faces numerous challenges, contributing to high stillbirth rates, including limited access to quality prenatal and perinatal care, high prevalence of infectious diseases such as malaria and HIV, poor maternal nutrition, and insufficient health infrastructure [4, 5]. Studies underscore the critical need for interventions to reduce stillbirths. Lawn et al. highlight that stillbirth rates in SSA have shown minimal improvement over the past decade, despite global health initiatives aimed at improving maternal and child health [4, 6]. The persistence of high stillbirth rates in the region indicates systemic issues in healthcare delivery and the need for targeted interventions to address the specific causes of stillbirths in this context [5, 7].

Although there is no a regional based study in SSA, numerous specific studies have identified various risk factors for stillbirth and neonatal mortality, including prematurity, previous perinatal death history, inadequate tetanus toxoid immunization, and insufficient iron supplementation [8, 9]. Furthermore, factors influencing stillbirth outcomes include parity [10–12], maternal age [10, 13], maternal education [14, 15], attendance of antenatal care visits (ANC) [12, 13, 16], presence of a skilled birth attendant [11], previous history of perinatal mortality [11, 13, 17], low family income, birth interval [16, 18], the total number of children under five [17], access to participation in decision-making [17], conception during teenage years [19], place of delivery [9], residency [13], and access to clean water supply [20].

Despite the significant burden of stillbirths in SSA, there are notable gaps in the literature regarding the specific factors contributing to this issue and the effectiveness of interventions. Most existing research is concentrated on high-income countries, with limited data on the unique challenges faced in SSA [4, 5, 7]. Additionally, there is a lack of comprehensive data on the determinants of stillbirths in the region, including the role of maternal education, access to healthcare, and traditional birth practices [21, 22].

The use of National Demographic and Health Surveys (DHS) data aligns with the Sustainable Development Goals (SDGs), particularly Goal 3, which aims to ensure healthy lives and promote well-being for all at all ages. One of the targets under this goal is to reduce neonatal mortality to at least as low as 12 per 1000 live births by 2030, a target that cannot be achieved without addressing the issue of stillbirths. By leveraging DHS data, policymakers and health practitioners can develop evidence-based strategies to reduce stillbirths and contribute to the achievement of the SDGs [22–24]. To support the achievement of SDG Goal 3, this study aimed to evaluate the prevalence and associated factors of stillbirths in SSA from 2016 to 2023.

## Methods

### Study design, setting, and period

This study relied on comprehensive, community-level surveys conducted across multiple countries. Governments and health organizations utilize a variety of data sources to guide healthcare planning, program implementation, and evaluation efforts in developing nations. The primary source of this crucial data is typically the routine health information system, which collects data from health facilities as well as population-based surveys [25]. We used data from the DHS of 27 SSA countries  conducted in a population-based cross-sectional study every five years in developing countries. This study used recent DHS data from SSA countries between 2016 and 2023.

### Source and study population

The source population for this study included all pregnancies at least 28 weeks' gestation among reproductive-age women in SSA countries. Whereas, the study population was drawn from selected enumeration areas within 27 SSA countries.

### Sampling size determination and sampling method

The DHS used a multi-stage, randomized sampling approach in each participating country. First, the survey teams randomly selected census areas, ensuring representation from both urban and rural regions within each administrative region. Then, they systematically chose individual households from within these selected areas to interview. The target participants were men and women of reproductive age living in these sampled households. After compiling the data from the various countries using statistical software, the researchers determined the final sample size based on the availability of the key outcome variable across the 27 SSA countries included in the study. In total, the analysis encompassed a weighted sample of 212,194 pregnancies of at least 28 weeks' gestation among reproductive age women, collected within the five years preceding the survey. This comprehensive, multi-stage sampling approach allowed the researchers to gather representative health data at both the community and national levels across SSA countries.

### Data sources

The analysis utilized secondary data from DHS conducted across SSA nations between 2016 and 2023. Specifically, the researchers accessed the DHS Birth Records (BR) databases for this study. To ensure that the sample data were representative of the target populations in these SSA countries, the researchers applied the appropriate sampling weights provided by DHS. This weighting process accounted for the unequal probabilities of household selection, thereby restoring the data's representativeness. By applying these weighted values, the analysis was able to generate findings that can be reliably generalized to the broader populations of the studied SSA nations, rather than being limited by potential biases in the sampling methodology. In summary, the secondary data analysis leveraged the robust DHS databases for SSA, using sound weighting techniques to produce representative and generalizable conclusions about the studied health outcomes [23].

### Variables of the study

#### Outcome variable

The outcome variable of this study was stillbirths. Stillbirths are defined as the number of fetal deaths in pregnancies lasting seven or more months during the five-year period preceding the survey. The stillbirth mortality rate was calculated as the number of stillbirths (numerator) divided by the total number of pregnancies meeting the criteria (denominator), multiplied by 1000. Then, the stillbirth mortality rate was categorized as Yes = 1 if the mother had experienced a stillbirth during her lifetime, and no = 0 otherwise.

#### Explanatory variables

Variables like maternal age, age at first birth, maternal education, marital status, wealth, mass media exposure, birth order, children ever born, under five children, history of abortion, birth interval in months, antenatal care visit, modern contraception, maternal employment, decision maker on women’s healthcare access, distance to health facility, source of drinking water, toilet facility types, health insurance, sex of household head, residence, country income, sub regions, and country literacy rate were the independent variables included in the analysis.

### Operational definitions

#### Country literacy rate

Evidence from the World Bank and World Population Review indicates that developed nations have an average literacy rate of more than 90%, while least developed nations have an average literacy rate of only 65% [26, 27]. Using this as a baseline, the researchers divided the countries' literacy rates into low (65% or below) and high (above the average 65%).

#### Country’s income level

The variable 'country income' was calculated using the World Bank classification, which divides countries into low income, lower middle income, and upper middle income categories [28].

#### Subregion

Based on their geographic location within the continent, the subregions were classified as follows: East Africa, West Africa, and Southern/Central Africa.

#### Source of drinking water

Source of drinking water was classified using the DHS guideline [23]. Improved source of drinking water: Households or de jure population using piped water into a dwelling or yard/plot, public taps/standpipes, water piped to a neighbor's property, tube wells or boreholes, protected wells, protected springs, rainwater colletion, tanker trucks or carts with small tanks, or bottled water are considered to have an improved source. Unimproved source of drinking water: Households or de jure population using unprotected wells, unprotected springs, surface water (rivers, dams, lakes, ponds, streams, canals, irrigation channels), or other unspecified sources are considered to have an unimproved source.

#### Toilet facility types

Improved toilet facility types include flush toilets connected to a piped sewer system, septic tank, pit latrine, or an unknown destination;ventilated improved pit (VIP) latrines; pit latrines with slabs; and composting toilets. On the other hand, unimproved toilet facility types include flush toilets that discharge to an unspecified location, pit latrines without slabs or open pits, bucket toilets, hanging toilets/latrines, and other unspecified types.

#### Mass media exposure

This variable was derived from three possible media sources: watching television, listening to the radio, and reading magazines/books. Women who were exposed to at least one of these media sources were recoded as having mass media exposure. Those who were not exposed to any of the three media sources were recoded as having no mass media exposure.

#### Birth interval

The birth interval, defined as the duration of time between consecutive live births, is typically classified as: very short (less than 18 months), short (18–24 months), optimal (25–59 months or 2–5 years), and long (60 months or 5 years and above).  Very short and short intervals are associated with increased health risks for the mother and infant, while optimal intervals allow sufficient time for maternal recovery and promote maternal and child health. Long intervals may potentially have some implications as well. This standardized classification scheme facilitates comprehensive research into the factors influencing birth spacing and its associated impacts.

### Data management process, and analysis

The researchers utilized R 4.4.0 software and Microsoft Excel 2019 to combine and analyze the data collected across the 27 SSA countries. To ensure the sample data accurately represented the true populations of these nations, the researchers weighted the data using the code “v005/1000000”. This weighting process accounted for the fact that children within the same cluster or sampling area tend to be more similar to each other than to children in other clusters. Applying these weights helped generate trustworthy standard errors and statistical estimates. The descriptive analysis involved cross-tabulations, as well as calculations of frequencies and percentages. This allowed the researchers to explore the patterns and distributions within the data in a clear and objective manner. By carefully preparing the data through recoding and weighting, and then conducting thorough descriptive analyses, the researchers were able to produce findings that reliably reflect the health characteristics and trends across the studied SSA populations [29]. A multivariable multilevel logistic regression model can account for the lack of independence across nested data layers [30, 31].

To determine the individual- and community-level factors for stillbirths among all pregnancies in the past five years before the survey. A two-stage multivariable multilevel logistic regression models were employed. Four models were used in this multilevel study. In the first model, there were no explanatory variables (null model); in the second model, there were only individual-level variables; in the third model, there were only community-level variables; and in the fourth model, there were both individual- and community-level variables. Adjusted odds ratios (AORs) with 95% confidence intervals (CIs) were used to present the fixed effects for results. Statistical significance was defined as a P value less than 0.05. The median odds ratio (MOR), proportionate change in variance (PCV), intra cluster correlation (ICC), log-likelihood ratio (LLR), and deviance were used as random effects measures to evaluate variation in childhood stillbirths across clusters. Deviance information criteria (DIC) and Akaike's information criterion (AIC) were used to compare models. The model with lower DIC and AIC values was deemed to be more accurate [32]. Before proceeding to the analysis, each dependent variable was assessed for variance, inflation factors, and tolerance.

### Ethical considerations and data set access

The study was conducted after obtaining a permission letter from https://www.dhsprogram.com/data/available-datasets.cfm on an online request to access DHS data from SSA countries after reviewing the submitted brief descriptions of the survey to the DHS program. The datasets were used with the utmost confidence. This study was done based on secondary data from SSA countries DHS. Issues related to informed consent, confidentiality, anonymity, and privacy of the study participants are already done ethically by the DHS office. There was no patient or public involvement in this study.

## Results

### Sociodemographic characteristic of participants

The majority of participants were aged between 35 and 49 years, accounting for 67.60% (143,442) of the sample. A significant portion of the participants had their first birth before the age of 20, making up 62.47% (132,564) of the group. Regarding education, 45.04% (95,571) of the participants were not educated, which was the largest group within the education variable. Most participants were married, comprising 71.33% (151,359) of the sample. In terms of economic status, the poorest individuals represented the largest wealth category at 22.48% (47,693). More than half of the participants (54.60% (115,851) did not have mass media exposure. When looking at birth order, the highest proportion of participants had their second or third child, making up 39.07% (82,904). A large majority of the participants 72.53% (153,902) had children under the age of five. Regarding their reproductive history, 80.80% (171,446) of the participants reported no history of abortion. The optimal birth interval of 24–59 months was reported by 43.15% (91,558) of the participants. Decisions on healthcare were predominantly made by others rather than the women themselves, as indicated by 81.43% (172,799) of the participants. The vast majority of participants (91.13% (193,382)) did not have health insurance. Most participants lived in rural areas, accounting for 68.31% (144,955). The participants predominantly came from low-income countries (54.49% (115,618)). Nearly half (101,980 (48.06%)) of them were from West African region (Table 1).

**Table 1:** Sociodemographic, maternal and child health related characteristics of participants in sub–Saharan Africa

Stillbirth mortality	Frequency (weighted)	Percentage
Variables		
*Maternal age*		
15–24	3,125	1.47
25–34	65,625	30.93
35–49	143,442	67.60
*Age at first birth*		
< 20	132,564	62.47
≥ 20	79,630	37.53
*Maternal education*		
Not educated	95,571	45.04
Primary	76,279	35.95
Secondary/higher	40,344	19.01
*Marital status*		
Not married	5,042	2.38
Married	151,359	71.33
Divorced/widowed	55,794	26.29
*Wealth*		
Poorest	47,693	22.48
Poorer	45,785	21.58
Middle	44,520	20.98
Richer	40,973	19.31
Richest	33,223	15.66
*Mass media exposure*		
No	115,851	54.60
Yes	96,343	45.40
*Birth order*		
1st	55,379	26.10
2nd or 3rd	82,904	39.07
4th and above	73,911	34.83
*Children ever born*		
< 3	14,899	7.02
3–5	91,123	42.94
> 5	106,173	50.04
*Under five children*		
No	58,292	27.47
Yes	153,902	72.53
*History of abortion*		
No	171,446	80.80
Yes	40,749	19.20
*Birth interval in months*		
Very short	17,146	8.08
Short	31,258	14.73
Optimal	91,558	43.15
Long	72,233	34.04
*At least one antenatal care visit*		
No	2,330	1.10
Yes	209,864	98.90
*Modern contraception*		
No	142,185	67.01
Yes	70,009	32.99
*Maternal employment*		
No	46,151	21.75
Yes	166,043	78.25
*Woman is decision maker on healthcare*		
No	172,799	81.43
Yes	39,395	18.57
*Distance to health facility*		
No problem	129,656	61.10
Big problem	82,538	38.90
*Source of drinking water*		
Not improved	82,285	38.78
Improved	129,909	61.22
*Toilet facility type*		
Not improved	106,458	50.17
Improved	105,736	49.83
*Health insurance*		
No	193,382	91.13
Yes	18,812	8.87
*Sex of household head*		
Male	158,448	74.67
Female	53,746	25.33
*Residence*		
Urban	67,239	31.69
Rural	144,955	68.31
*Country income*		
Low	115,618	54.49
Lower middle	93,647	44.13
Upper middle	2,929	1.38
*Country literacy rate*		
Low	115,006	54.20
High	97,188	45.80
*Sub regions*		
East	78,639	37.06
West	101,980	48.06
South/central	31,575	14.88

### Pooled prevalence of stillbirths in sub-Saharan Africa

**Fig. 1:**
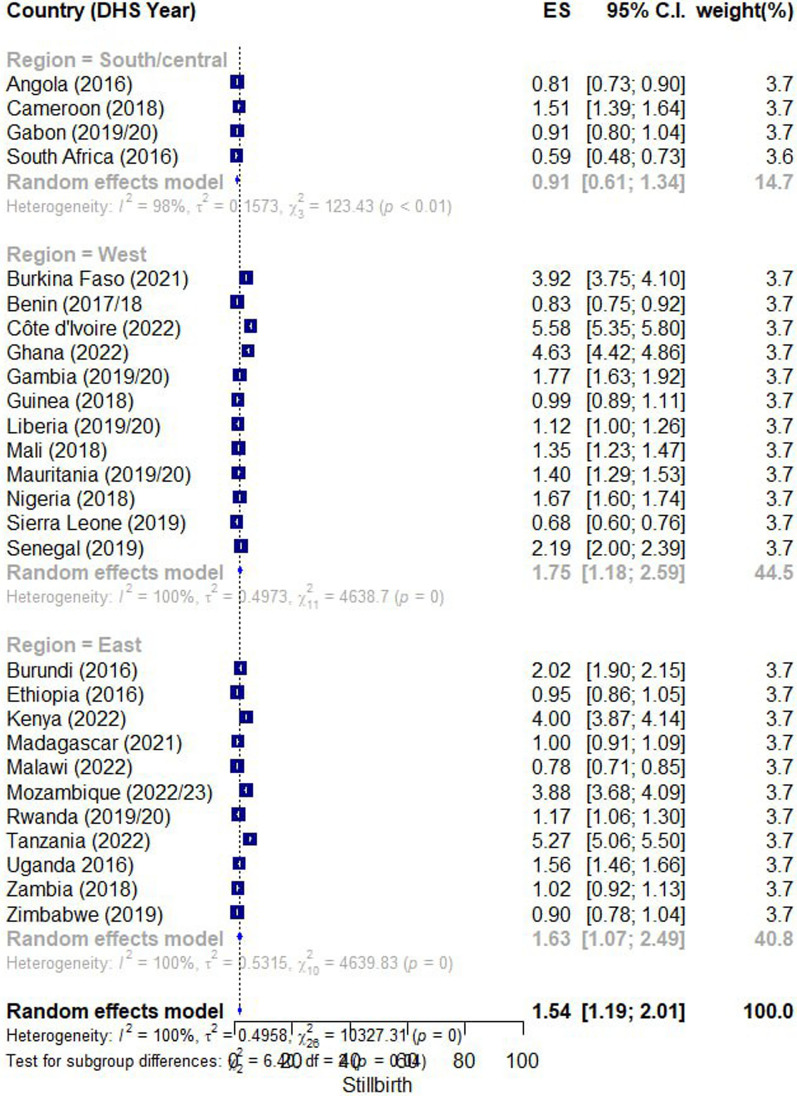
Pooled prevalence of stillbirths in 27 SSA countries from 2016 to 2023

The forest plot shows a meta-analysis of stillbirth rates across various SSA countries, categorized by region. The overall random effects model yielded a pooled prevalence of 15.4 per 1000 or 1.54% per100 [95% CI 1.19, 2.01]. The analysis showed a very high heterogeneity, indicated by I^2^ = 100%, (tau^2^ = 0.4958), and (chi^2^ = 13,027.31) (p = 0.0001). Additionally, the test for subgroup differences revealed a statistically significant difference in stillbirth rates among the regions, with (chi^2^ = 6.20), df = 2 (*p* = 0.045). The high heterogeneity (I^2^ = 100%) suggests substantial variability in stillbirth rates within and between regions, highlighting the diverse epidemiological landscape of stillbirths across SSA countries. The test for subgroup differences indicates a statistically significant difference in stillbirth rates among the regions (*p* = 0.045) (Fig. 1).

### Random effects analyses

Table 2 presents a comparison of four statistical models (Null model, Model I, Model II, and Model III) used to analyze stillbirths in SSA regions. The models were evaluated through several key parameters: community-level variance, ICC, MOR, PCV, LLR, DIC, and AIC.

**Table 2:** Random parameters and model comparison for stillbirth mortality in sub–Saharan Africa

Random parameters and model comparison	Null model	Model I	Model II	Model III
Community level variance	0.30	0.29	0.28	0.25
ICC	8.11	8.03	7.76	7.65
MOR	3.73	3.67	3.62	3.17
PCV	Reference	3.33	6.67	16.67
LLR	− 21,540	− 21,282	− 21,386	− 21,185
DIC	43,080	42,564	42,772	42,370
AIC	43,083	42,615	42,784	42,429

The community-level variance, which measures the variation in stillbirth mortality rates at the community level, showed a decreasing trend from 0.30 in the Null model to 0.25 in Model III. This reduction indicates that incorporating additional predictors in Models I, II, and III could help explain some of the community-level variation in stillbirths. The ICC, which reflects the proportion of total variance attributable to differences between communities, remained relatively stable across the models, starting at 8.11 in the Null model and decreasing slightly to 7.7 in Model III. This stability suggests that a substantial portion of the total variance in stillbirths could be attributed to community differences.

The MOR, which quantifies the median value of the odds ratio between two randomly chosen individuals from different communities, decreased marginally from 3.73 in the Null model to 3.17 in Model III. This indicates a modest reduction in between-community heterogeneity in stillbirth risk as more predictors were included. The PCV, representing the percentage reduction in community-level variance compared to the Null model, increased from 3.33% in Model I to 16.67% in Model III, showing that Model III was the most effective in reducing unexplained community-level variance.

The LLR, a measure of model fit where higher (less negative) values indicate a better fit, improved from −21,540 in the Null model to −21,185 in Model III. This improvement suggests that Model III had the best fit among the four models. The DIC and AIC, both used to compare model goodness-of-fit with lower values indicating better performance, also decreased progressively, with Model III showing the lowest values (42,370 for DIC and 42,429 for AIC). This further confirmed that Model III provided the best fit for the data. In summary, the comparison indicates that Model III was the most effective in explaining the variance in stillbirth at the community level among the four models tested. It achieved the best fit, comfirmed by the lowest DIC and AIC values and the highest LLR, while also demonstrating significant reductions in community-level variance and maintaining a stable ICC. These suggest that Model III successfully incorporated critical predictors, thereby improving the understanding and estimation of stillbirth mortality in SSA (Table 2).

### Factors associated with stillbirth in SSA

Several significant variables, marked with an asterisk (*), were compared against a constant reference category (denoted by 1) and demonstrated a statistically significant association with stillbirths. Mothers aged 25–34 years had higher odds of experiencing stillbirths compared to those aged 15–24 years, with an AOR of 1.22 (95% CI 1.14–1.27) in Model III. This risk increased further for mothers aged 35–49 with an AOR of 1.40 (95% CI 1.32–1.48). Mothers with primary education had lower odds of experiencing stillbirths than those with no education, with an AOR of 0.83 (95% CI 0.79–0.85). The odds decreased even further for mothers with secondary or higher education, with an AOR of 0.62 (95% CI 0.59–0.64). Compared to the poorest category, poorer individuals had slightly lower odds of experiencing stillbirths (AOR of 0.91, 95% CI 0.88–0.95). This trend continued across the middle (AOR of 0.92, 95% CI 0.88–0.95) and richer (AOR of 0.96, 95% CI 0.91–0.99) categories. The richest individuals had significantly lower odds, with an AOR of 0.85 (95% CI 0.80–0.90). Individuals with access to mass media had lower odds of experiencing stillbirths with an AOR of 0.82 (95% CI 0.75–0.89) compared to those without access. Married individuals had higher odds of experiencing stillbirths compared to unmarried individuals, with an AOR of 1.98 (95% CI 1.78–2.20). Divorced or widowed individuals had even higher odds, with an AOR of 2.01 (95% CI 1.80–2.23). Individuals who attended ANC visits had  higher odds with an AOR of 1.37 (95% CI 1.21–1.54). Women who had their first birth before age 20 had higher odds with an AOR of 1.09 (95% CI 1.05–1.12). Access to improved drinking water sources was linked to lower stillbirth odds, with an AOR of 0.89 (95% CI 0.87–0.92). Short birth intervals were associated with an AOR of 1.06 (95% CI 1.02–1.14), optimal intervals with an AOR of 1.14 (95% CI 1.08–1.21), and long intervals with the highest odds, with an AOR of 1.27 (95% CI 1.20–1.36). Individuals who did not perceive distance to health facilities as a problem had lower odds of stillbirth, with an AOR of 0.92 (95% CI 0.89–0.94). Compared to first births, second or third births had higher odds of experiencing stillbirths, with an AOR of 1.08 (95% CI 1.03–1.14). Residence in rural areas was associated with higher stillbirth odds compared to urban areas, with an AOR of 1.16 (95% CI 1.12–1.20). Countries classified as lower-middle-income had higher odds of experiencing stillbirths compared to low-income countries, with an AOR of 1.54 (95% CI 1.50–1.59). Conversely, upper-middle-income countries had significantly lower odds, with an AOR of 0.48 (95% CI 0.38–0.60). Regions with low literacy rates had higher odds, with an AOR of 1.31 (95% CI 1.27–1.35) (Table 3).

**Table 3:** Individual and contextual-level factors associated with stillbirth mortality in sub–Saharan Africa

Stillbirth mortality in SSA	Null model	Model I, AOR 95%	Model II, AOR 95%	Model III, AOR 95%
Variables				
*Maternal age*				
15–24		1		1
25–34		1.22 (1.16,1.29)		1.22 (1.14, 1.27)*
35–49		1.42 (1.34,1.51)		1.40 (1.32, 1.48)*
*Maternal education*				
Not educated		1		1
Primary		0.89 (0.86,0.92)		0.83 (0.79, 0.85)*
Secondary/higher		0.71 (0.67,0.74)		0.62 (0.59, 0.64)*
*Maternal working*				
Not working		1		1
Working		0.98 (0.95,1.02)		1.01 (0.97, 1.04)
*Wealth index*				
Poorest		1		1
Poorer		0.88 (0.85,0.92)		0.91 (0.88, 0.95)*
Middle		0.85 (0.82,0.88)		0.92 (0.88, 0.95)*
Richer		0.83 (0.80,0.87)		0.96 (0.91, 0.99)*
Richest		0.68 (0.64,0.71)		0.85 (0.80, 0.90)*
*Mass media*				
No		1		1
Yes		0.78 (0.72,0.85)		0.82 (0.75, 0.89)*
*Marital status*				
Unmarried		1		1
Married		2.08 (1.88,2.32)		1.98 (1.78, 2.20)*
Divorced/widowed		2.13 (1.91,2.37)		2.01 (1.80, 2.23)*
*ANC visit*				
No		1		1
Yes		1.36 (1.21,1.54)		1.37 (1.21, 1.54)*
*Age at first birth*				
< 20		1.08 (1.05,1.11)		1.09 (1.05, 1.12)*
≥ 20		1		1
*Source drinking water*				
Not improved		1		1
Improved		0.89 (0.86,0.91)		0.89 (0.87, 0.92)*
*Birth interval months*				
Very short		1		1
Short		1.06 (0.99,1.13)		1.06 (1.02, 1.14)*
Optimal		1.13 (1.07,1.19)		1.14 (1.08, 1.21)*
Long		1.27 (1.18,1.35)		1.27 (1.20, 1.36)*
*Distance to health facility*				
Problem		1		1
No problem		0.89 (0.86,0.92)		0.92 (0.89, 0.94)*
*Health insurance*				
No		1		1
Yes		1.19 (1.14,1.24)		1.04 (0.99, 1.08)
*Decision maker on healthcare*				
Women herself		1		1
Others		0.97 (0.93,1.01)		0.99 (0.96, 1.03)
*Birth order*				
First		1		1
2nd or 3rd		1.08 (1.03,1.14)		1.08 (1.03, 1.14)*
≥ 4th		0.99 (0.94,1.05)		0.98 (0.94, 1.04)
*Residence*				
Urban			1	1
Rural			1.31 (1.27,1.35)	1.16 (1.12, 1.20)*
*Country income*				
Low			1	1
Lower middle			1.52 (1.48,1.57)	1.54 (1.50, 1.59)*
Upper middle			0.34 (0.27,0.42)	0.48 (0.38, 0.60)*
Country literacy rate			1	1
High			1	1
Low			1.19 (1.16,1.23)	1.31 (1.27, 1.35)*

*Statistically significant variables in the final model

## Discussion

The overarching objective of this study was to comprehensively examine the pooled prevalence of stillbirths and the key factors associated with it across SSA countries after the implementation of the SDG to reduce child mortality by improving reproductive, maternal, and child health throughout the globe, especially in developing countries. By analyzing recent DHS data from 27 countries in the region, the researchers sought to gain a thorough understanding of this critical public health issue. The random effects model revealed that there was a significant variation in stillbirth rates in SSA. The pooled prevalence was 1.54% per 100 and there was a high level of heterogeneity (I^2^ = 100%). This variability indicates that there were different epidemiological contexts in the region, which means that targeted interventions are needed. The significant subgroup differences (*p* = 0.045) also highlight the importance of developing region-specific strategies to address the underlying factors contributing to stillbirths. These findings demonstrate the need for comprehensive public health policies that are tailored to the unique challenges faced by different regions in order to effectively reduce stillbirth rates and achieve SDG.

Maternal age significantly affected the likelihood of stillbirths in this study, a finding consistent with previous literature from East Africa [33], Zambia [6], and Australia [34]. Different age groups face varying levels of risk. Women in their late 20s to early 30s may have a higher risk due to declined natural fertility and reduced egg quality [35]. However, the risk becomes even more pronounced after the age of 35. Older women often experience more chronic health conditions, such as preeclampsia, blood clotting disorders, twin pregnancies, and an increased risk of Down syndrome [36]. These factors contribute to a higher chance of stillbirths compared to younger mothers. In addition, older women may have lifestyle factors that impact pregnancy, such as delaying childbearing and higher stress levels. For older women, the risk of stillbirths increases even further. As a woman gets older, the quality and quantity of her eggs decline rapidly after the age of 35. This raises the odds of chromosomal abnormalities and problems with the placenta, both of which can lead to stillbirths. Furthermore, older mothers are more prone to complications such as gestational diabetes, preeclampsia, and restricted fetal growth, all of which are linked to higher rates of stillbirths.

Mothers with primary education had lower odds of experiencing stillbirths than those with no education in this study. The odds decreased further for those with secondary or higher education. This finding aligns with research conducted in Bangladesh [37], East Africa [33], and Nepal [38]. One potential reason for this could be that literate mothers have a heightened understanding of potential problems and are more inclined to seek prenatal care early on. Additionally, they may have a better grasp of and are able to adhere to the guidance provided by healthcare professionals effectively. Media exposure decreased the likelihood of stillbirths in this study, which aligns with previous research findings [33, 39]. This may be attributed to the fact that mass media is an influential tool for increasing awareness, knowledge, and positive behaviors related to maternal healthcare utilization [40]. Specifically, when reproductive-age women are exposed to mass media, they are more likely to utilize family planning services and ANC services, and deliver in health facilities. Consequently, this increased utilization can help reduce the risk of stillbirths [41].

Socioeconomic status was a significant factor affecting the risk of stillbirths in this study. It seems that expectant mothers from economically disadvantaged households are more likely to experience stillbirths compared to those from wealthier families. Previous research conducted in East Africa [33], Uganda [42], Ethiopia [18], and Nepal [43] have confirmed this relationship, as they found similar patterns linking household wealth to stillbirth outcomes. The reasons behind this are likely related to the fact that women from wealthier backgrounds tend to have higher levels of health awareness and engage in better health-seeking behaviors. They are more likely to understand the importance of proper nutrition, regular antenatal care, and avoid harmful practices during pregnancy. On the other hand, women living in poverty may have limited access to essential maternal healthcare services, even when these services are offered free of charge. The indirect costs associated with transportation, time away from work, and other logistical barriers can prevent poorer women from fully utilizing these critical interventions, which may contribute to their increased risk of stillbirths. Ultimately, the disparities in stillbirth rates between different socioeconomic groups highlight the need to address the various social determinants of maternal and child health. This includes ensuring equitable access to quality healthcare and empowering women across all socioeconomic levels.

Births from,divorced or widowed women were found to have an increased risk of stillbirths in this study, as highlighted in previous studies [27, 33, 44, 55]. This could be attributed to the fact that divorced or widowed women often face significant stress and financial constraints in accessing maternal healthcare services, including the additional costs for transportation [44, 45]. Moreover, these women generally lack social support and are more likely to experience distress, which further increases their risk of stillbirths.

ANC visits were associated with higher stillbirth odds in this study. Women who have ANC follow-up may reveal higher odds of stillbirths due to several factors. High-risk pregnancies are more likely to be identified and closely monitored through ANC visits, leading to a higher report of complications and stillbirths. Detection and reporting biases also play a role, as regular ANC follow-up increases the likelihood of identifying issues that might go unnoticed in women without ANC. Furthermore, women attending ANC visits often have preexisting conditions or complications that inherently increase the risk of stillbirths, irrespective of the care they receive. Additionally, the quality and timing of ANC visits can impact outcomes, with late initiation or lower-quality care being less effective in preventing stillbirths. Socioeconomic and environmental factors, such as chronic health conditions, lifestyle factors, and stressors, also contribute to the observed higher odds of stillbirths among women who attend ANC follow-ups [46–48]. These findings suggest that women attending ANC visits may represent a higher-risk group due to underlying health issues contributing to their increased stillbirth risk.

Access to improved drinking water sources was found to be linked to lower stillbirth odds in this study. Assessments of the disease burden caused by inadequate water, sanitation, and hygiene (WASH) have primarily focused on deaths and illnesses related to diarrhea [49]. Although the number of deaths from diarrhea has decreased, it still accounts for 10% of all child deaths [50]. There is solid evidence linking WASH to various other health issues, including respiratory infections, infections caused by parasitic worms and diseases resulting from chemical contamination of water [51]. Further studies are recommended to prove its effect on stillbirth and perinatal mortality. Birth intervals significantly affected stillbirth odds, with both short, and long intervals associated with higher risks compared to optimal intervals in this study. This finding challenges the conventional view that shorter intervals are riskier due to factors like inadequate maternal recovery [33]. However, maternal health, presence of chronic disease, terminated pregnancy, and other genetic and health service utilization might be raised as possible factors [52, 53]. Further research with better study design and long follow up studies might uncover this dilemma [54, 55]. Long intervals might also be associated with complications similar to those experienced by first-time mothers. Further research with refined designs and longer follow-ups is needed to better understand these dynamics and develop targeted interventions [52, 53]. Our results underscore the complexity of how birth intervals influence stillbirth risk and call for a more detailed investigation.

Distance to health facilities was found to be another important factor. Women who did not perceive distance to health facilities as a barrier had lower odds of experiencing stillbirths. Although previous literature does not provide direct evidence that reduced distance to health facilities is linked to lower odds of stillbirths, it might reduce the possibility of accessing maternal and child health services on time [56, 57]. Studies have found that travel time from a woman's residential area to the main referral hospital was a strong predictor of stillbirth risk. Women living beyond 1 h away from a hospital had a 12-fold higher likelihood of stillbirths compared to those within 15 min [56]

Compared to first births, the odds of stillbirths were found to be higher for second or third births due to several factors. Women who had experienced a stillbirth in a previous pregnancy faced an elevated risk of stillbirths in subsequent pregnancies, especially if the prior stillbirth occurred at an early gestational age. The risk decreased if the previous stillbirth happened later in pregnancy [58–60]. The higher risk of recurrent stillbirth is often linked to underlying medical conditions such as hypertension, diabetes, and placental abruption. Women with a history of stillbirths are more likely to have these risk factors compared to those who have only experienced live births [59]. The risk of stillbirth recurrence is highest for women who had a stillbirth in their first pregnancy and decreases with each subsequent pregnancy [59, 61]. This suggests that the gestational age of the previous stillbirth plays a significant role, as the risk of stillbirth appears to increase with each subsequent pregnancy, especially if the prior stillbirth occurred during a critical period of fetal development [60, 61]. In this study, there was a significant association between place of residence and stillbirths. This study indicates that women living in rural areas had a higher incidence of stillbirths compared to women in urban areas. This finding aligns with similar studies conducted in Ethiopia [62] and East Africa [33]. The reason behind this correlation might be the limited access to healthcare and information about pregnancy, labor, and delivery. Additionally, delays in seeking medical attention may also contribute to the higher rate of stillbirths among rural women.

### Strengths and limitations

The presented study has several notable strengths. One of its main strengths is the use of nationally representative and large-sample DHS data. This extensive dataset allows for a more accurate and generalizable representation of stillbirth mortality rates and their determinants across SSA countries, which improves the reliability and applicability of the findings. Additionally, the study utilized a comprehensive approach by considering individual/household, community, and country-level factors as potential contributors to stillbirth mortality. This multi-level analysis provides valuable insights into the complex factors influencing stillbirth mortality rates. Moreover, the holistic perspective gained from this analysis can also contribute to progress towards relevant SDG targets. However, the study have some limitations. It lacks data on clinical, cultural, time as independent variables, and prospective factors that may be associated with stillbirths. The absence of these variables in the DHS dataset restricts the study's ability to fully explore the impact of medical factors on stillbirth rates. Future research should aim to incorporate these additional data sources to facilitate a more comprehensive analysis. Addressing these gaps through further research will contribute to a more thorough understanding of the determinants of stillbirth mortality.

### Recommendations and the way forward

Programs to improve educational attainment, especially for women, should be prioritized. This includes promoting secondary and higher education to reduce the risk of stillbirths. Efforts to elevate economic status, particularly among the poorest populations, can significantly reduce stillbirth rates. This involves creating employment opportunities and providing financial support to low-income families. Expanding access to mass media can disseminate critical health information, potentially reducing stillbirth rates. Strengthening ANC services and encouraging early and regular ANC visits can help identify and manage potential complications early. Special attention should be given to improving healthcare access and quality in rural areas, including enhancing transportation and health infrastructure. Additionally, providing a targeted support and counseling for married and divorced/widowed women to mitigate their higher risk of stillbirths. Furthermore, programs aimed at delaying the age of first birth and supporting young mothers can reduce stillbirth rates. Education on family planning and reproductive health is crucial. Ensuring access to improved drinking water and sanitation facilities can contribute to reducing stillbirth rates. Family planning services should emphasize the importance of optimal birth intervals to reduce the risk of stillbirths. Finally, initiatives to improve literacy rates and provide health education can empower women with the knowledge to make informed health decisions. By implementing a comprehensive approach addressing various social, economic, and healthcare factors, the goal of reducing stillbirth rates can be more effectively achieved.

To effectively reduce stillbirths in SSA, a multi-faceted approach is required. Policymakers should focus on improving education and economic conditions, particularly for women and low-income families. Health systems must be strengthened to provide comprehensive maternal and child healthcare services, especially in rural and underserved areas. Collaboration between governments, non-governmental organizations, and communities is essential to implement these recommendations effectively. Continued research and data collection are necessary to monitor progress and identify emerging trends. By addressing the identified factors associated with stillbirths, significant strides can be made in improving maternal and child health outcomes in SSA.

## Conclusions

The study highlights that stillbirths are a significant concern in SSA, with rates remaining alarmingly high and falling significantly short of achieving Every Newborn Action Plan target by 2030. By analyzing individual and contextual-level factors, the final model, which controls for confounders, highlights several significant variables associated with stillbirths. Stillbirths in SSA are driven by a complex interplay of older maternal age, better education, economic status, mass media exposure, marital status, antenatal care, age at first birth, drinking water access, birth interval, distance to health facilities, birth order, rural residence, country income levels, and literacy rates. Addressing these factors through targeted interventions can help mitigate the high stillbirth rates in the region. To enhance the understanding of stillbirths in SSA, we recommend focusing on improving education and healthcare access. Specifically, targeted educational programs for expectant mothers and community health workers, alongside improved access to quality healthcare services, should be prioritized to address and mitigate the risk factors associated with stillbirths.

## Data Availability

All data concerning this study are accommodated and presented in this document. The datasets used and/or analyzed during the current study available from the corresponding author on reasonable request.

## References

[1] Rantakallio P, Oja H. Perinatal risk for infants of unmarried mothers, over a period of 20 years. Early Human Dev. 1990;22(3):157–69. 10.1016/0378-3782(90)90182-i 2397715

[2] World Health Organization. Every Newborn: an action plan to end preventable deaths: https://www.who.int/publications/i/item/9789241507448. 2014.

[3] Unicef. A neglected tragedy: the global burden of stillbirths: report of the UN Inter-agency Group for Child Mortality Estimation, 2020. 2020.

[4] Lawn JE, Blencowe H, Waiswa P, Amouzou A, Mathers C, Hogan D, et al. Stillbirths: rates, risk factors, and acceleration towards 2030. The Lancet. 2016;387(10018):587–603. 10.1016/S0140-6736(15)00837-5 26794078

[5] Blencowe H, Cousens S, Jassir FB, Say L, Chou D, Mathers C, et al. National, regional, and worldwide estimates of stillbirth rates in 2015, with trends from 2000: a systematic analysis. Lancet Glob Health. 2016;4(2):e98–108. 26795602 10.1016/S2214-109X(15)00275-2

[6] Stringer EM, Vwalika B, Killam WP, Giganti MJ, Mbewe R, Chi BH, et al. Determinants of stillbirth in Zambia. Obstet Gynecol. 2011;117(5):1151–9. 21508755 10.1097/AOG.0b013e3182167627

[7] Aminu M, Bar-Zeev S, White S, Mathai M, van Den Broek N. Understanding cause of stillbirth: a prospective observational multi-country study from sub-Saharan Africa. BMC Pregnancy Childbirth. 2019;19:1–10. 31801488 10.1186/s12884-019-2626-7 PMC6894270

[8] Goba GK, Tsegay H, Gebregergs GB, Mitiku M, Kim KA, Alemayehu M. A facility-based study of factors associated with perinatal mortality in Tigray, northern Ethiopia. Int J Gynecol Obstet. 2018;141(1):113–9. 10.1002/ijgo.12438 29318613

[9] Roro EM, Sisay MM, Sibley LM. Determinants of perinatal mortality among cohorts of pregnant women in three districts of North Showa zone, Oromia Region, Ethiopia: community based nested case control study. BMC Public Health. 2018;18:1–11. 10.1186/s12889-018-5757-2 PMC6052561 30021557

[10] Arach AAO, Tumwine JK, Nakasujja N, Ndeezi G, Kiguli J, Mukunya D, et al. Perinatal death in Northern Uganda: incidence and risk factors in a community-based prospective cohort study. Glob Health Action. 2021;14(1):1859823. 33446087 10.1080/16549716.2020.1859823 PMC7832989

[11] Debelew GT. Magnitude and determinants of perinatal mortality in Southwest Ethiopia. J Pregnancy. 2020. 10.1155/2020/6859157. 33029401 10.1155/2020/6859157 PMC7528145

[12] Seyoum E, Bekele A, Tsegaye AT, Birhanu S. Magnitude and determinants of adverse perinatal outcomes in Tefera Hailu Memorial Hospital, Sekota Town, Northern Ethiopia. Global Pediatric Health. 2021;8(2333):2333794X211015524. 34036123 10.1177/2333794X211015524 PMC8127752

[13] Nwokoro UU, Dahiru T, Olorukooba A, Daam CK, Waziri HS, Adebowale A, et al. Determinants of perinatal mortality in public secondary health facilities, Abuja municipal area council, Federal Capital Territory, Abuja, Nigeria. Pan African Medical Journal. 2020. 10.11604/pamj.2020.37.114.17108. 33425147 10.11604/pamj.2020.37.114.17108 PMC7755356

[14] Martins EF, Rezende EM, Almeida MCDM, Lana FCF. Perinatal mortality and socio-spatial inequalities. Rev Lat Am Enfermagem. 2013;21:1062–70. 24142214 10.1590/S0104-11692013000500008

[15] Usynina AA, Grjibovski AM, Krettek A, Odland JØ, Kudryavtsev AV, Anda EE. Risk factors for perinatal mortality in Murmansk County, Russia: a registry-based study. Glob Health Action. 2017;10(1):1270536. 28156197 10.1080/16549716.2017.1270536 PMC5328313

[16] Geda A, Shemsu S, Debalke R. Determinants of perinatal mortality in public hospitals of Iluu Abbaa Boor Oromia Region, South West Ethiopia, 2019: unmatched case–control study. Res Rep Neonatol. 2021:57–66.

[17] Hossain MB, Kanti Mistry S, Mohsin M, Rahaman Khan MH. Trends and determinants of perinatal mortality in Bangladesh. PLoS ONE. 2019;14(8):e0221503. 31442258 10.1371/journal.pone.0221503 PMC6707592

[18] Jena BH, Biks GA, Gelaye KA, Gete YK. Magnitude and trend of perinatal mortality and its relationship with inter-pregnancy interval in Ethiopia: a systematic review and meta-analysis. BMC Pregnancy Childbirth. 2020;20:1–13. 10.1186/s12884-020-03089-2 PMC7389567 32727403

[19] Siddalingappa H, Kulkarni P, Ashok N. Prevalence and factors influencing perinatal mortality in rural Mysore, India. J Clin Diagn Res JCDR. 2013;7(12):2796. 24551640 10.7860/JCDR/2013/6367.3761 PMC3919272

[20] Organization WH. Neonatal and perinatal mortality: country, regional and global estimates: World Health Organization; 2006.

[21] McClure EM, Saleem S, Goudar SS, Garces A, Whitworth R, Esamai F, et al. Stillbirth 2010–2018: a prospective, population-based, multi-country study from the Global Network. Reprod Health. 2020;17:1–9. 33256783 10.1186/s12978-020-00991-y PMC7706249

[22] Bhutta ZA. Counting stillbirths and achieving accountability: a global health priority. PLoS Med. 2017;14(8):e1002364. 28763442 10.1371/journal.pmed.1002364 PMC5538629

[23] Croft T, Marshall AM, Allen CK, Arnold F, Assaf S, Balian S, et al. Guide to DHS Statistics: DHS-7 (version 2). Rockville, MD: ICF. 2020.

[24] United Nations Department of Economic and Social Affairs PD. Sustainable Development Goals: https://sdgs.un.org/goals.

[25] Boerma JT, Sommerfelt AE. Demographic and health surveys (DHS): contributions and limitations. World Health Stat Q. 1993;46(4):222–6. 8017081

[26] Soenens B, Vansteenkiste M, Beyers W. Parenting adolescents. Handbook of parenting: Routledge; 2019. p. 111–67.

[27] The World Bank. Literacy rate (%): https://genderdata.worldbank.org/en/indicator/se-adt. 2024.

[28] World Bank. World Bank Group country classifications by income level: https://blogs.worldbank.org/en/opendata/new-world-bank-group-country-classifications-income-level-fy24. 2024.

[29] Macro O. Central statistical agency: Ethiopia demographic and health survey 2005. ORC Macro, Calverton, Maryland, USA. 2006;3:6–59.

[30] Goldstein H. Multilevel statistical models. Wiley; 2011.

[31] Larsen K, Merlo J. Appropriate assessment of neighborhood effects on individual health: integrating random and fixed effects in multilevel logistic regression. Am J Epidemiol. 2005;161(1):81–8. 15615918 10.1093/aje/kwi017

[32] Zuur AF, Ieno EN, Walker NJ, Saveliev AA, Smith GM. Mixed effects models and extensions in ecology with R. Springer; 2009.

[33] Tesema GA, Tessema ZT, Tamirat KS, Teshale AB. Prevalence of stillbirth and its associated factors in East Africa: generalized linear mixed modeling. BMC Pregnancy Childbirth. 2021;21(1):414. 34078299 10.1186/s12884-021-03883-6 PMC8173886

[34] Gordon A, Raynes-Greenow C, McGeechan K, Morris J, Jeffery H. Risk factors for antepartum stillbirth and the influence of maternal age in New South Wales Australia: a population based study. BMC Pregnancy Childbirth. 2013;13:1–10. 23324309 10.1186/1471-2393-13-12 PMC3552834

[35] Mills TA, Lavender T. Advanced maternal age. Obstet Gynaecol Reprod Med. 2011;21(4):107–11.

[36] Reddy UM, Ko C-W, Willinger M. Maternal age and the risk of stillbirth throughout pregnancy in the United States. Am J Obstet Gynecol. 2006;195(3):764–70. 16949411 10.1016/j.ajog.2006.06.019

[37] Nahar S, Rahman A, Nasreen HE. Factors influencing stillbirth in B angladesh: a case–control study. Paediatr Perinat Epidemiol. 2013;27(2):158–64. 23374060 10.1111/ppe.12026

[38] Kc A, Nelin V, Wrammert J, Ewald U, Vitrakoti R, Baral GN, Målqvist M. Risk factors for antepartum stillbirth: a case-control study in Nepal. BMC Pregnancy Childbirth. 2015;15:1–10. 26143456 10.1186/s12884-015-0567-3 PMC4491416

[39] Yakoob MY, Menezes EV, Soomro T, Haws RA, Darmstadt GL, Bhutta ZA. Reducing stillbirths: behavioural and nutritional interventions before and during pregnancy. BMC Pregnancy Childbirth. 2009;9:1–34. 19426466 10.1186/1471-2393-9-S1-S3 PMC2679409

[40] Olayinka OA, Achi OT, Amos AO, Chiedu EM. Awareness and barriers to utilization of maternal health care services among reproductive women in Amassoma community. Bayelsa State Int J Nurs Midwifery. 2014;6(1):10–5.

[41] Joshi C, Torvaldsen S, Hodgson R, Hayen A. Factors associated with the use and quality of antenatal care in Nepal: a population-based study using the demographic and health survey data. BMC Pregnancy Childbirth. 2014;14:1–11. 24589139 10.1186/1471-2393-14-94 PMC3943993

[42] Kujala S, Waiswa P, Kadobera D, Akuze J, Pariyo G, Hanson C. Trends and risk factors of stillbirths and neonatal deaths in Eastern Uganda (1982–2011): a cross-sectional, population-based study. Trop Med Int Health. 2017;22(1):63–73. 27910181 10.1111/tmi.12807

[43] Kc A, Wrammert J, Ewald U, Clark RB, Gautam J, Baral G, et al. Incidence of intrapartum stillbirth and associated risk factors in tertiary care setting of Nepal: a case-control study. Reprod Health. 2016;13:1–11. 27581467 10.1186/s12978-016-0226-9 PMC5007702

[44] August E, Salihu H, Weldeselasse H, Biroscak B, Mbah A, Alio A. Infant mortality and subsequent risk of stillbirth: a retrospective cohort study. BJOG Int J Obstetr Gynaecol. 2011;118(13):1636–45. 10.1111/j.1471-0528.2011.03137.x 21933338

[45] Olds DL, Henderson Jr CR, Kitzman HJ, Eckenrode JJ, Cole RE, Tatelbaum RC. Prenatal and infancy home visitation by nurses: Recent findings. Fut Children. 1999;44–65. 10414010

[46] Berhe T, Modibia LM, Sahile AT, Tedla GW. Does quality of antenatal care influence antepartum stillbirth in Hossana City, South Ethiopia? PLOS Global Public Health. 2023;3(1):e0001468. 36963030 10.1371/journal.pgph.0001468 PMC10021135

[47] Purbey A, Nambiar A, Choudhury DR, Vennam T, Balani K, Agnihotri SB. Stillbirth rates and its spatial patterns in India: an exploration of HMIS data. Lancet Reg Health-Southeast Asia. 2023;9. 10.1016/j.lansea.2022.100116 PMC10306056 37383033

[48] Kasa GA, Woldemariam AY, Adella A, Alemu B. The factors associated with stillbirths among sub-saharan African deliveries: a systematic review and meta-analysis. BMC Pregnancy Childbirth. 2023;23(1):835. 38049743 10.1186/s12884-023-06148-6 PMC10696713

[49] Guerrant RL, Kosek M, Moore S, Lorntz B, Brantley R, Lima AA. Magnitude and impact of diarrheal diseases. Arch Med Res. 2002;33(4):351–5. 12234524 10.1016/s0188-4409(02)00379-x

[50] Liu L, Johnson HL, Cousens S, Perin J, Scott S, Lawn JE, et al. Global, regional, and national causes of child mortality: an updated systematic analysis for 2010 with time trends since 2000. Lancet. 2012;379(9832):2151–61. 22579125 10.1016/S0140-6736(12)60560-1

[51] Ziegelbauer K, Speich B, Mäusezahl D, Bos R, Keiser J, Utzinger J. Effect of sanitation on soil-transmitted helminth infection: systematic review and meta-analysis. PLoS Med. 2012;9(1):e1001162. 22291577 10.1371/journal.pmed.1001162 PMC3265535

[52] Goldenberg RL, McClure EM, MacGuire ER, Kamath BD, Jobe AH. Lessons for low-income regions following the reduction in hypertension-related maternal mortality in high-income countries. Int J Gynecol Obstet. 2011;113(2):91–5. 10.1016/j.ijgo.2011.01.002 21349517

[53] Goldenberg RL, Saleem S, Pasha O, Harrison MS, Mcclure EM. Reducing stillbirths in low-income countries. Acta Obstet Gynecol Scand. 2016;95(2):135–43. 26577070 10.1111/aogs.12817

[54] Conde-Agudelo A, Rosas-Bermúdez A, Kafury-Goeta AC. Birth spacing and risk of adverse perinatal outcomes: a meta-analysis. JAMA. 2006;295(15):1809–23. 16622143 10.1001/jama.295.15.1809

[55] DaVanzo J, Hale L, Razzaque A, Rahman M. Effects of interpregnancy interval and outcome of the preceding pregnancy on pregnancy outcomes in Matlab, Bangladesh. BJOG Int J Obstetr Gynaecol. 2007;114(9):1079–87. 10.1111/j.1471-0528.2007.01338.x PMC2366022 17617195

[56] Banke-Thomas A, Avoka CK-O, Gwacham-Anisiobi U, Benova L. Influence of travel time and distance to the hospital of care on stillbirths: a retrospective facility-based cross-sectional study in Lagos, Nigeria. BMJ Glob Health. 2021;6(10):007052. 10.1136/bmjgh-2021-007052 PMC8496383 34615663

[57] Wariri O, Onuwabuchi E, Alhassan JAK, Dase E, Jalo I, Laima CH, et al. The influence of travel time to health facilities on stillbirths: a geospatial case-control analysis of facility-based data in Gombe, Nigeria. PLoS ONE. 2021;16(1):e0245297. 33411850 10.1371/journal.pone.0245297 PMC7790442

[58] Al Khalaf S, Kublickiene K, Kublickas M, Khashan AS, Heazell AE. Risk of stillbirth and adverse pregnancy outcomes in a third pregnancy when an earlier pregnancy has ended in stillbirth. Acta Obstet Gynecol Scand. 2024;103(1):111–20. 37891707 10.1111/aogs.14705 PMC10755120

[59] Surkan PJ, Stephansson O, Dickman PW, Cnattingius S. Previous preterm and small-for-gestational-age births and the subsequent risk of stillbirth. N Engl J Med. 2004;350(8):777–85. 14973215 10.1056/NEJMoa031587

[60] Nijkamp JW, Ravelli AC, Groen H, Erwich JJH, Mol BWJ. Stillbirth and neonatal mortality in a subsequent pregnancy following stillbirth: a population-based cohort study. BMC Pregnancy Childbirth. 2022;22(1):11. 34983439 10.1186/s12884-021-04355-7 PMC8725424

[61] Lamont K, Scott NW, Jones GT, Bhattacharya S. Risk of recurrent stillbirth: systematic review and meta-analysis. bmj. 2015;350. 10.1136/bmj.h3080 26109551

[62] Wolde J, Haile D, Paulos K, Alemayehu M, Adeko AC, Ayza A. Prevalence of stillbirth and associated factors among deliveries attended in health facilities in Southern Ethiopia. PLoS ONE. 2022;17(12):e0276220. 36512623 10.1371/journal.pone.0276220 PMC9746959

